# Ethyl 4-(furan-2-yl)-6-methyl-2-oxo-1,2,3,4-tetra­hydro­pyrimidine-5-carboxyl­ate

**DOI:** 10.1107/S1600536810040456

**Published:** 2010-10-20

**Authors:** Hong-Yun Wang

**Affiliations:** a College of Chemistry and Chemical Technology, Binzhou University, Binzhou 256600, Shandong, People’s Republic of China

## Abstract

The asymmetric unit of the title compound, C_12_H_14_N_2_O_4_, contains two independent mol­ecules. In one independent mol­ecule, the furanyl fragment is rotationally disordered between two orientations in a 0.625 (6):0.375 (6) ratio. In the crystal, inter­molecular pyrimidine–pyrimidinone N—H⋯O hydrogen bonds link the mol­ecules into centrosymmetric tetra­mers, which are further associated into ribbons extending in [010] *via* weak inter­molecular pyrimidine–carboxyl N—H⋯O hydrogen bonds.

## Related literature

The Biginelli reaction is the most important procedure in the synthesis of 3,4-dihydro­pyrimidin-2-(1*H*)-ones, see: Biginelli (1893 ▶). For related structures, see: Nizam Mohideen *et al.* (2008 ▶); Qing-Fang *et al.* (2007 ▶).
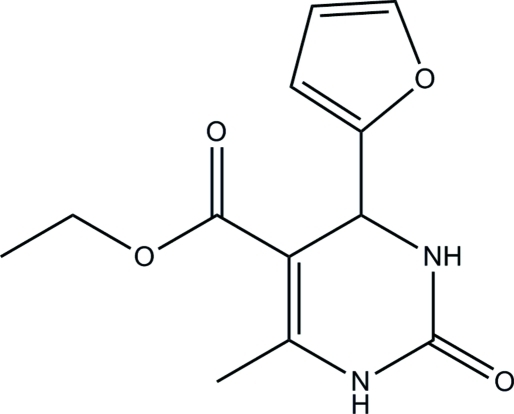


## Experimental

### 

#### Crystal data

C_12_H_14_N_2_O_4_
*M* *_r_* = 250.25
Monoclinic, 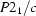

*a* = 12.1720 (14) Å
*b* = 13.3180 (15) Å
*c* = 17.116 (2) Å
α = 90°
β = 118.300 (2)°
γ = 90°
*V* = 2443.0 (5) Å^3^
*Z* = 8
Mo *K*α radiation
μ = 0.10 mm^−1^
*T* = 298 K
0.48 × 0.45 × 0.17 mm

#### Data collection

Bruker SMART APEX CCD area-detector diffractometer
Absorption correction: multi-scan (*SADABS*; Sheldrick, 1996 ▶) *T*_min_ = 0.952, *T*_max_ = 0.983
11604 measured reflections
4298 independent reflections
1943 reflections with *I* > 2σ(*I*)
*R*_int_ = 0.069

#### Refinement

*R*[*F* ^2^ > 2σ(*F* ^2^)] = 0.071
*wR*(*F* ^2^) = 0.237
*S* = 0.94
4298 reflections
338 parameters
H-atom parameters constrained
Δρ_max_ = 0.30 e Å^−3^
Δρ_min_ = −0.23 e Å^−3^

### 

Data collection: *SMART* (Bruker, 2007 ▶); cell refinement: *SAINT* (Bruker, 2007 ▶); data reduction: *SAINT*; program(s) used to solve structure: *SHELXS97* (Sheldrick, 2008 ▶); program(s) used to refine structure: *SHELXL97* (Sheldrick, 2008 ▶); molecular graphics: *SHELXTL* (Sheldrick, 2008 ▶); software used to prepare material for publication: *SHELXTL*.

## Supplementary Material

Crystal structure: contains datablocks I, global. DOI: 10.1107/S1600536810040456/cv2771sup1.cif

Structure factors: contains datablocks I. DOI: 10.1107/S1600536810040456/cv2771Isup2.hkl

Additional supplementary materials:  crystallographic information; 3D view; checkCIF report

## Figures and Tables

**Table 1:** Hydrogen-bond geometry (Å, °)

*D*—H⋯*A*	*D*—H	H⋯*A*	*D*⋯*A*	*D*—H⋯*A*
N1—H1⋯O1^i^	0.86	2.12	2.924 (4)	156
N2—H2⋯O5^ii^	0.86	2.02	2.851 (4)	162
N3—H3⋯O4^iii^	0.86	2.38	3.077 (4)	138
N4—H4⋯O1^iv^	0.86	2.10	2.952 (4)	174

Symmetry codes: (i) 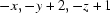; (ii) 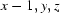; (iii) 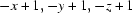; (iv) 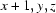.

**Fig. 1.**
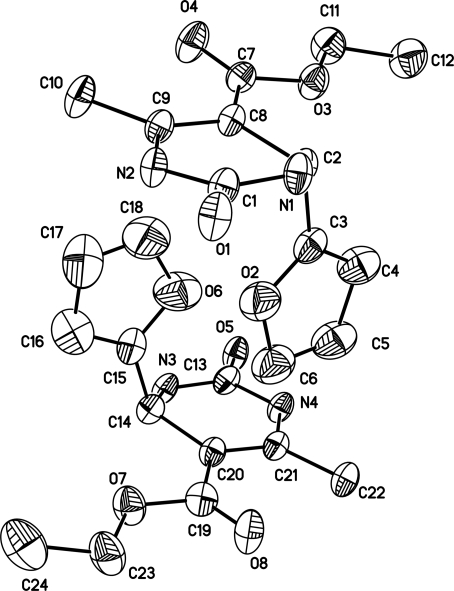
The content of asymmetric unit of the title compound showing the atomic numbering scheme and 30% probability displacement ellipsoids. Only major components of the disordered atoms are shown.

## supplementary crystallographic information

### Comment

Biginelli reaction is a well known multicomponent reaction involving a one-pot cyclocondensation of an aldehyde, β-ketoester and urea/thiourea. It is the most important procedure in the synthesis of 3,4-dihydropyrimidin-2-(1*H*)-ones (Biginelli, 1893). Herewith we report the crystal structure of the title compound, (I), obtained by the three-component reaction of furfuraldehyde, acetoacetate and urea.

In (I) (Fig. 1), the dihydropyrimidinone rings adopt flattened boat conformation. The asymmetric unit contains two independent molecules. In one independent molecule, the furanyl fragment is rotationally disordered in a ratio 0.625 (6):0.375 (6). The bond lengths an angles are normal and comparable to the values observed in similar compounds (Nizam Mohideen *et al.*, 2008; Qing-Fang *et al.*, 2007) The dihedral angles between the furan rings (C3—C6/O2, C15—C18/O6) and the mean planes of the dihydropyrimidinone rings (N1/C1/N2/C9/C8, N3/C13/N4/C21/C20) unit in two independent molecules are 88.79 (4) ° and 86.73 (2)°, respectively, indicating that the furan rings and the dihydropyrimidinone rings are nearly perpendicular.

In the crystal structure, intermolecular *N*—*H*···O_pyrimidinone_ hydrogen bonds (Table 1) link the molecules into centrosymmetric tetramers. Tetramers are further associated into ribbons extended in direction [010] *via* the weak intermolecular N—H···O_carboxyl_ hydrogen bonds (Table 1).

### Experimental

A mixture of ethylacetoacetate (0.5 mol), furfural (0.5 mol) and urea (0.6 mol) was refluxed in 50.0 ml of ethanol for 2.0 hrs. The reaction completion was monitored through thin layer chromatography and the reaction mixture was quenched in ice cold water. The precipitate obtained was filtered, dried and crystallized from methanol to obtain the title compound.

### Refinement

All H atoms were placed in geometrically idealized positions (N—H 0.86 and C—H = 0.93–0.97 Å) and treated as riding on their parent atoms, with *U*_iso_(H) = 1.2U-1.5_eq_(C, N). Atoms C4, C5, C6, O2 were treated as disordered between two positions, with refined occupancies of 0.375 (6) and 0.625 (6).

### Crystal data

**Table:**

C_12_H_14_N_2_O_4_	*F*(000) = 1056
*M**_r_* = 250.25	*D*_x_ = 1.361 Mg m^−^^3^
Monoclinic, *P*2_1_/*c*	Mo *K*α radiation, λ = 0.71073 Å
*a* = 12.1720 (14) Å	Cell parameters from 1664 reflections
*b* = 13.3180 (15) Å	θ = 2.3–22.3°
*c* = 17.116 (2) Å	µ = 0.10 mm^−^^1^
β = 118.300 (2)°	*T* = 298 K
*V* = 2443.0 (5) Å^3^	Block, yellow
*Z* = 8	0.48 × 0.45 × 0.17 mm

### Data collection

**Table:**

Bruker SMART APEX CCD area-detector diffractometer	4298 independent reflections
Radiation source: fine-focus sealed tube	1943 reflections with *I* > 2σ(*I*)
graphite	*R*_int_ = 0.069
φ and ω scans	θ_max_ = 25.0°, θ_min_ = 1.9°
Absorption correction: multi-scan (*SADABS*; Sheldrick, 1996)	*h* = −14→14
*T*_min_ = 0.952, *T*_max_ = 0.983	*k* = −14→15
11604 measured reflections	*l* = −20→19

### Refinement

**Table:**

Refinement on *F*^2^	Primary atom site location: structure-invariant direct methods
Least-squares matrix: full	Secondary atom site location: difference Fourier map
*R*[*F*^2^ > 2σ(*F*^2^)] = 0.071	Hydrogen site location: inferred from neighbouring sites
*wR*(*F*^2^) = 0.237	H-atom parameters constrained
*S* = 0.94	*w* = 1/[σ^2^(*F*_o_^2^) + (0.1244*P*)^2^] where *P* = (*F*_o_^2^ + 2*F*_c_^2^)/3
4298 reflections	(Δ/σ)_max_ = 0.001
338 parameters	Δρ_max_ = 0.30 e Å^−^^3^
0 restraints	Δρ_min_ = −0.23 e Å^−^^3^

### Special details

**Table:**

Geometry. All e.s.d.'s (except the e.s.d. in the dihedral angle between two l.s. planes) are estimated using the full covariance matrix. The cell e.s.d.'s are taken into account individually in the estimation of e.s.d.'s in distances, angles and torsion angles; correlations between e.s.d.'s in cell parameters are only used when they are defined by crystal symmetry. An approximate (isotropic) treatment of cell e.s.d.'s is used for estimating e.s.d.'s involving l.s. planes.
Refinement. Refinement of *F*^2^ against ALL reflections. The weighted *R*-factor *wR* and goodness of fit *S* are based on *F*^2^, conventional *R*-factors *R* are based on *F*, with *F* set to zero for negative *F*^2^. The threshold expression of *F*^2^ > σ(*F*^2^) is used only for calculating *R*-factors(gt) *etc*. and is not relevant to the choice of reflections for refinement. *R*-factors based on *F*^2^ are statistically about twice as large as those based on *F*, and *R*- factors based on ALL data will be even larger.

### Fractional atomic coordinates and isotropic or equivalent isotropic displacement parameters (Å^2^)

**Table:**

	*x*	*y*	*z*	*U*_iso_*/*U*_eq_	Occ. (<1)
N1	0.0677 (3)	0.8975 (2)	0.4632 (2)	0.0478 (9)	
H1	0.0351	0.9555	0.4437	0.057*	
N2	0.0574 (3)	0.7467 (2)	0.5219 (2)	0.0479 (8)	
H2	0.0468	0.7135	0.5609	0.057*	
N3	0.8736 (3)	0.6124 (2)	0.7072 (2)	0.0499 (9)	
H3	0.8988	0.5511	0.7182	0.060*	
N4	0.8981 (3)	0.7698 (2)	0.6635 (2)	0.0497 (8)	
H4	0.9196	0.8075	0.6321	0.060*	
O1	−0.0199 (2)	0.88634 (18)	0.55364 (19)	0.0574 (8)	
O2	0.3239 (6)	0.8613 (5)	0.5879 (5)	0.086 (2)	0.625 (6)
C4'	0.3192 (18)	0.9228 (13)	0.5941 (13)	0.086 (2)	0.375 (6)
H4'	0.2672	0.9425	0.6173	0.103*	0.375 (6)
O3	0.2203 (2)	0.7669 (2)	0.32562 (17)	0.0565 (8)	
O4	0.1728 (3)	0.6108 (2)	0.3447 (2)	0.0686 (9)	
O5	0.9657 (3)	0.63333 (18)	0.6200 (2)	0.0600 (8)	
O6	0.6054 (4)	0.6411 (3)	0.5976 (3)	0.1107 (13)	
O7	0.7327 (3)	0.7358 (2)	0.85737 (19)	0.0690 (9)	
O8	0.7471 (3)	0.8936 (2)	0.8223 (2)	0.0780 (10)	
C1	0.0329 (3)	0.8474 (3)	0.5145 (3)	0.0456 (10)	
C2	0.1585 (3)	0.8589 (3)	0.4384 (3)	0.0429 (10)	
H2A	0.1438	0.8907	0.3826	0.052*	
C3	0.2873 (4)	0.8830 (3)	0.5074 (3)	0.0559 (11)	
C4	0.3859 (8)	0.9166 (7)	0.4951 (7)	0.079 (2)	0.625 (6)
H4A	0.3852	0.9349	0.4424	0.094*	0.625 (6)
C5	0.4870 (15)	0.9164 (9)	0.5815 (12)	0.086 (4)	0.625 (6)
H5	0.5680	0.9359	0.5966	0.103*	0.625 (6)
C6	0.448 (2)	0.8828 (11)	0.6403 (17)	0.091 (4)	0.625 (6)
H6	0.4943	0.8764	0.7016	0.109*	0.625 (6)
O2'	0.3875 (9)	0.8450 (8)	0.5162 (7)	0.079 (2)	0.375 (6)
C5'	0.459 (4)	0.924 (2)	0.636 (3)	0.091 (4)	0.375 (6)
H5'	0.5116	0.9619	0.6855	0.109*	0.375 (6)
C6'	0.492 (3)	0.8685 (16)	0.598 (2)	0.086 (4)	0.375 (6)
H6'	0.5725	0.8445	0.6184	0.103*	0.375 (6)
C7	0.1783 (3)	0.6992 (3)	0.3629 (2)	0.0461 (9)	
C8	0.1410 (3)	0.7470 (2)	0.4236 (2)	0.0408 (9)	
C9	0.0980 (3)	0.6952 (3)	0.4705 (2)	0.0425 (9)	
C10	0.0877 (4)	0.5845 (3)	0.4755 (3)	0.0621 (12)	
H10A	0.0024	0.5646	0.4395	0.093*	
H10B	0.1149	0.5652	0.5359	0.093*	
H10C	0.1390	0.5523	0.4542	0.093*	
C11	0.2614 (4)	0.7310 (3)	0.2650 (3)	0.0666 (12)	
H11A	0.1938	0.6969	0.2155	0.080*	
H11B	0.3299	0.6842	0.2946	0.080*	
C12	0.3030 (4)	0.8203 (4)	0.2328 (3)	0.0828 (15)	
H12A	0.2344	0.8660	0.2037	0.124*	
H12B	0.3312	0.7988	0.1918	0.124*	
H12C	0.3700	0.8533	0.2823	0.124*	
C13	0.9139 (3)	0.6678 (3)	0.6603 (3)	0.0473 (10)	
C14	0.7910 (3)	0.6476 (3)	0.7409 (3)	0.0477 (10)	
H14	0.8183	0.6161	0.7989	0.057*	
C15	0.6605 (4)	0.6159 (3)	0.6829 (3)	0.0588 (12)	
C16	0.5810 (5)	0.5651 (4)	0.7005 (4)	0.0925 (17)	
H16	0.5969	0.5387	0.7552	0.111*	
C17	0.4691 (6)	0.5587 (5)	0.6215 (6)	0.113 (2)	
H17	0.3961	0.5281	0.6141	0.136*	
C18	0.4851 (6)	0.6031 (6)	0.5604 (6)	0.126 (3)	
H18	0.4254	0.6087	0.5011	0.151*	
C19	0.7596 (4)	0.8057 (3)	0.8129 (3)	0.0563 (11)	
C20	0.8035 (3)	0.7603 (3)	0.7555 (2)	0.0452 (9)	
C21	0.8509 (3)	0.8162 (3)	0.7128 (2)	0.0444 (9)	
C22	0.8605 (4)	0.9280 (3)	0.7135 (3)	0.0591 (12)	
H22A	0.7799	0.9562	0.6756	0.089*	
H22B	0.9178	0.9474	0.6924	0.089*	
H22C	0.8901	0.9524	0.7729	0.089*	
C23	0.6852 (5)	0.7708 (4)	0.9158 (3)	0.0831 (15)	
H23A	0.6148	0.8154	0.8839	0.100*	
H23B	0.7496	0.8074	0.9654	0.100*	
C24	0.6464 (5)	0.6833 (5)	0.9477 (4)	0.110 (2)	
H24A	0.5780	0.6508	0.8987	0.165*	
H24B	0.6206	0.7041	0.9903	0.165*	
H24C	0.7150	0.6373	0.9753	0.165*	

### Atomic displacement parameters (Å^2^)

**Table:**

	*U*^11^	*U*^22^	*U*^33^	*U*^12^	*U*^13^	*U*^23^
N1	0.047 (2)	0.0349 (18)	0.072 (2)	0.0098 (14)	0.037 (2)	0.0113 (15)
N2	0.058 (2)	0.0331 (19)	0.065 (2)	0.0014 (15)	0.0384 (19)	0.0075 (14)
N3	0.053 (2)	0.0363 (18)	0.074 (2)	0.0055 (14)	0.041 (2)	0.0085 (15)
N4	0.056 (2)	0.0355 (19)	0.068 (2)	0.0017 (15)	0.038 (2)	0.0059 (15)
O1	0.0673 (18)	0.0414 (16)	0.088 (2)	0.0067 (13)	0.0563 (18)	0.0067 (14)
O2	0.068 (3)	0.082 (5)	0.086 (4)	−0.010 (4)	0.019 (3)	0.002 (5)
C4'	0.068 (3)	0.082 (5)	0.086 (4)	−0.010 (4)	0.019 (3)	0.002 (5)
O3	0.0712 (19)	0.0502 (17)	0.0654 (18)	−0.0097 (13)	0.0466 (17)	−0.0051 (13)
O4	0.089 (2)	0.0406 (18)	0.097 (2)	−0.0088 (15)	0.061 (2)	−0.0133 (15)
O5	0.0728 (19)	0.0429 (17)	0.091 (2)	0.0006 (14)	0.0604 (19)	0.0001 (14)
O6	0.077 (3)	0.116 (3)	0.105 (3)	−0.018 (2)	0.015 (3)	0.006 (2)
O7	0.083 (2)	0.070 (2)	0.076 (2)	−0.0077 (16)	0.055 (2)	−0.0078 (16)
O8	0.100 (3)	0.052 (2)	0.105 (3)	−0.0020 (17)	0.066 (2)	−0.0134 (17)
C1	0.041 (2)	0.041 (2)	0.064 (3)	0.0000 (17)	0.032 (2)	0.0031 (19)
C2	0.044 (2)	0.037 (2)	0.055 (2)	−0.0022 (17)	0.030 (2)	0.0033 (17)
C3	0.049 (3)	0.041 (2)	0.074 (3)	−0.001 (2)	0.027 (3)	−0.004 (2)
C4	0.058 (3)	0.084 (6)	0.090 (5)	−0.003 (5)	0.031 (4)	−0.011 (5)
C5	0.052 (4)	0.090 (11)	0.100 (9)	−0.006 (8)	0.024 (6)	−0.015 (9)
C6	0.068 (6)	0.081 (14)	0.095 (6)	−0.007 (10)	0.016 (5)	−0.005 (11)
O2'	0.058 (3)	0.084 (6)	0.090 (5)	−0.003 (5)	0.031 (4)	−0.011 (5)
C5'	0.068 (6)	0.081 (14)	0.095 (6)	−0.007 (10)	0.016 (5)	−0.005 (11)
C6'	0.052 (4)	0.090 (11)	0.100 (9)	−0.006 (8)	0.024 (6)	−0.015 (9)
C7	0.043 (2)	0.045 (3)	0.053 (2)	−0.0040 (18)	0.025 (2)	−0.0023 (19)
C8	0.040 (2)	0.033 (2)	0.053 (2)	−0.0022 (16)	0.025 (2)	−0.0002 (16)
C9	0.041 (2)	0.037 (2)	0.057 (2)	−0.0012 (16)	0.029 (2)	−0.0004 (17)
C10	0.079 (3)	0.035 (2)	0.091 (3)	−0.009 (2)	0.055 (3)	−0.005 (2)
C11	0.075 (3)	0.070 (3)	0.071 (3)	−0.005 (2)	0.048 (3)	−0.004 (2)
C12	0.086 (4)	0.094 (4)	0.088 (4)	−0.023 (3)	0.057 (3)	−0.005 (3)
C13	0.044 (2)	0.039 (2)	0.064 (3)	0.0000 (17)	0.029 (2)	0.0050 (18)
C14	0.049 (2)	0.044 (2)	0.059 (3)	0.0016 (18)	0.033 (2)	0.0051 (18)
C15	0.054 (3)	0.049 (3)	0.081 (4)	−0.005 (2)	0.038 (3)	−0.007 (2)
C16	0.077 (4)	0.106 (5)	0.107 (5)	−0.027 (3)	0.054 (4)	−0.013 (3)
C17	0.068 (4)	0.110 (6)	0.161 (7)	−0.021 (4)	0.054 (5)	−0.043 (5)
C18	0.069 (5)	0.127 (6)	0.123 (7)	−0.007 (4)	−0.002 (5)	−0.019 (5)
C19	0.046 (3)	0.059 (3)	0.066 (3)	−0.005 (2)	0.028 (2)	−0.003 (2)
C20	0.038 (2)	0.041 (2)	0.057 (2)	0.0002 (17)	0.022 (2)	−0.0021 (18)
C21	0.038 (2)	0.037 (2)	0.058 (3)	−0.0007 (16)	0.022 (2)	−0.0011 (17)
C22	0.063 (3)	0.039 (2)	0.080 (3)	−0.0017 (19)	0.037 (3)	0.000 (2)
C23	0.103 (4)	0.090 (4)	0.082 (3)	−0.009 (3)	0.065 (3)	−0.013 (3)
C24	0.122 (5)	0.140 (6)	0.106 (4)	−0.030 (4)	0.084 (4)	−0.017 (4)

### Geometric parameters (Å, °)

**Table:**

N1—C1	1.322 (4)	O2'—C6'	1.41 (3)
N1—C2	1.454 (4)	C5'—C6'	1.17 (6)
N1—H1	0.8600	C5'—H5'	0.9300
N2—C1	1.365 (4)	C6'—H6'	0.9300
N2—C9	1.379 (4)	C7—C8	1.462 (5)
N2—H2	0.8600	C8—C9	1.339 (5)
N3—C13	1.343 (5)	C9—C10	1.485 (5)
N3—C14	1.454 (4)	C10—H10A	0.9600
N3—H3	0.8600	C10—H10B	0.9600
N4—C21	1.373 (4)	C10—H10C	0.9600
N4—C13	1.377 (4)	C11—C12	1.496 (6)
N4—H4	0.8600	C11—H11A	0.9700
O1—C1	1.241 (4)	C11—H11B	0.9700
O2—C3	1.263 (8)	C12—H12A	0.9600
O2—C6	1.37 (2)	C12—H12B	0.9600
C4'—C3	1.44 (2)	C12—H12C	0.9600
C4'—C5'	1.50 (5)	C14—C15	1.480 (5)
C4'—H4'	0.9300	C14—C20	1.518 (5)
O3—C7	1.338 (4)	C14—H14	0.9800
O3—C11	1.430 (4)	C15—C16	1.327 (6)
O4—C7	1.211 (4)	C16—C17	1.395 (8)
O5—C13	1.224 (4)	C16—H16	0.9300
O6—C15	1.330 (6)	C17—C18	1.294 (9)
O6—C18	1.387 (7)	C17—H17	0.9300
O7—C19	1.337 (5)	C18—H18	0.9300
O7—C23	1.449 (5)	C19—C20	1.455 (5)
O8—C19	1.201 (4)	C20—C21	1.349 (5)
C2—C3	1.484 (5)	C21—C22	1.494 (5)
C2—C8	1.509 (5)	C22—H22A	0.9600
C2—H2A	0.9800	C22—H22B	0.9600
C3—O2'	1.262 (10)	C22—H22C	0.9600
C3—C4	1.387 (10)	C23—C24	1.458 (7)
C4—C5	1.405 (18)	C23—H23A	0.9700
C4—H4A	0.9300	C23—H23B	0.9700
C5—C6	1.38 (3)	C24—H24A	0.9600
C5—H5	0.9300	C24—H24B	0.9600
C6—H6	0.9300	C24—H24C	0.9600
			
C1—N1—C2	122.9 (3)	H10A—C10—H10B	109.5
C1—N1—H1	118.6	C9—C10—H10C	109.5
C2—N1—H1	118.6	H10A—C10—H10C	109.5
C1—N2—C9	123.9 (3)	H10B—C10—H10C	109.5
C1—N2—H2	118.0	O3—C11—C12	107.3 (4)
C9—N2—H2	118.0	O3—C11—H11A	110.3
C13—N3—C14	125.2 (3)	C12—C11—H11A	110.3
C13—N3—H3	117.4	O3—C11—H11B	110.3
C14—N3—H3	117.4	C12—C11—H11B	110.3
C21—N4—C13	125.1 (3)	H11A—C11—H11B	108.5
C21—N4—H4	117.5	C11—C12—H12A	109.5
C13—N4—H4	117.5	C11—C12—H12B	109.5
C3—O2—C6	112.1 (12)	H12A—C12—H12B	109.5
C3—C4'—C5'	101 (2)	C11—C12—H12C	109.5
C3—C4'—H4'	129.5	H12A—C12—H12C	109.5
C5'—C4'—H4'	129.5	H12B—C12—H12C	109.5
C7—O3—C11	117.6 (3)	O5—C13—N3	124.2 (4)
C15—O6—C18	106.5 (5)	O5—C13—N4	120.8 (3)
C19—O7—C23	117.0 (4)	N3—C13—N4	115.0 (4)
O1—C1—N1	123.9 (4)	N3—C14—C15	111.5 (3)
O1—C1—N2	120.4 (3)	N3—C14—C20	110.5 (3)
N1—C1—N2	115.6 (3)	C15—C14—C20	112.6 (3)
N1—C2—C3	110.7 (3)	N3—C14—H14	107.3
N1—C2—C8	109.4 (3)	C15—C14—H14	107.3
C3—C2—C8	111.0 (3)	C20—C14—H14	107.3
N1—C2—H2A	108.5	C16—C15—O6	109.7 (5)
C3—C2—H2A	108.5	C16—C15—C14	131.2 (5)
C8—C2—H2A	108.5	O6—C15—C14	119.1 (4)
O2'—C3—O2	87.6 (7)	C15—C16—C17	107.0 (6)
O2'—C3—C4	44.8 (5)	C15—C16—H16	126.5
O2—C3—C4	110.9 (6)	C17—C16—H16	126.5
O2'—C3—C4'	104.6 (10)	C18—C17—C16	107.7 (6)
O2—C3—C4'	35.0 (6)	C18—C17—H17	126.2
C4—C3—C4'	101.9 (9)	C16—C17—H17	126.2
O2'—C3—C2	127.2 (6)	C17—C18—O6	109.1 (7)
O2—C3—C2	120.8 (5)	C17—C18—H18	125.4
C4—C3—C2	127.8 (6)	O6—C18—H18	125.4
C4'—C3—C2	124.4 (9)	O8—C19—O7	121.5 (4)
C3—C4—C5	103.2 (10)	O8—C19—C20	127.3 (4)
C3—C4—H4A	128.4	O7—C19—C20	111.3 (4)
C5—C4—H4A	128.4	C21—C20—C19	121.7 (4)
C6—C5—C4	109.5 (15)	C21—C20—C14	119.5 (3)
C6—C5—H5	125.2	C19—C20—C14	118.8 (3)
C4—C5—H5	125.2	C20—C21—N4	119.7 (3)
O2—C6—C5	104.1 (18)	C20—C21—C22	126.7 (3)
O2—C6—H6	127.9	N4—C21—C22	113.5 (3)
C5—C6—H6	127.9	C21—C22—H22A	109.5
C3—O2'—C6'	113.1 (15)	C21—C22—H22B	109.5
C6'—C5'—C4'	110 (3)	H22A—C22—H22B	109.5
C6'—C5'—H5'	125.2	C21—C22—H22C	109.5
C4'—C5'—H5'	125.2	H22A—C22—H22C	109.5
C5'—C6'—O2'	108 (3)	H22B—C22—H22C	109.5
C5'—C6'—H6'	125.9	O7—C23—C24	107.9 (4)
O2'—C6'—H6'	125.9	O7—C23—H23A	110.1
O4—C7—O3	121.3 (3)	C24—C23—H23A	110.1
O4—C7—C8	127.4 (3)	O7—C23—H23B	110.1
O3—C7—C8	111.2 (3)	C24—C23—H23B	110.1
C9—C8—C7	122.9 (3)	H23A—C23—H23B	108.4
C9—C8—C2	118.2 (3)	C23—C24—H24A	109.5
C7—C8—C2	118.9 (3)	C23—C24—H24B	109.5
C8—C9—N2	119.1 (3)	H24A—C24—H24B	109.5
C8—C9—C10	127.9 (3)	C23—C24—H24C	109.5
N2—C9—C10	113.0 (3)	H24A—C24—H24C	109.5
C9—C10—H10A	109.5	H24B—C24—H24C	109.5
C9—C10—H10B	109.5		

### Hydrogen-bond geometry (Å, °)

**Table:**

*D*—H···*A*	*D*—H	H···*A*	*D*···*A*	*D*—H···*A*
N1—H1···O1^i^	0.86	2.12	2.924 (4)	156
N2—H2···O5^ii^	0.86	2.02	2.851 (4)	162
N3—H3···O4^iii^	0.86	2.38	3.077 (4)	138
N4—H4···O1^iv^	0.86	2.10	2.952 (4)	174

Symmetry codes: (i) −*x*, −*y*+2, −*z*+1; (ii) *x*−1, *y*, *z*; (iii) −*x*+1, −*y*+1, −*z*+1; (iv) *x*+1, *y*, *z*.
